# Kainate receptor subunit 1 (*GRIK1*) risk variants and GRIK1 deficiency were detected in the Indian ADHD probands

**DOI:** 10.1038/s41598-022-21948-0

**Published:** 2022-11-02

**Authors:** Mahasweta Chatterjee, Sharmistha Saha, Nilanjana Dutta, Swagata Sinha, Kanchan Mukhopadhyay

**Affiliations:** Manovikas Biomedical Research and Diagnostic Centre, Manovikas Kendra, 482, Madudah, Plot I-24, Sec.-J, E.M. Bypass, Kolkata, West Bengal 700107 India

**Keywords:** Cell biology, Developmental biology, Genetics, Molecular biology, Neuroscience, Biomarkers, Medical research

## Abstract

Executive dysfunctions caused by structural and functional abnormalities of the prefrontal cortex were reported in patients with Attention deficit hyperactivity disorder (ADHD). Owing to a higher expression of the glutamate ionotropic receptor kainate type subunit 1 (GluK1), encoded by the *GRIK1* gene, in brain regions responsible for learning and memory, we hypothesized that *GRIK1* might have a role in ADHD. *GRIK1* variants rs363504 and rs363538, affecting the receptor function, were analyzed by case–control and family-based methods to identify the association with ADHD. The impact of these variants on ADHD-associated traits and pharmacological intervention were also analyzed. GRIK1 expression was quantified in the peripheral blood. The probands and their fathers had a higher frequency of rs363504 ‘CC’ and rs363538 ‘CA’ genotypes. Family-based investigation revealed maternal over transmission of rs363504 ‘C’ and rs363538 ‘A’ alleles to the probands. Quantitative trait analysis exhibited an association of rs363504 ‘TT’ and rs363538 ‘AA’ genotypes with higher hyperactivity scores of the probands. In the presence of rs363504 ‘TT’ and rs363538 ‘CC’ genotypes, MPH treatment improved hyperactivity and inattention, respectively. GRIK1 expression was significantly downregulated in the probands. We infer that GRIK1 affects ADHD etiology, warranting further in-depth investigation involving a larger cohort and more functional variants.

## Introduction

One of the most prevalent neurobehavioral disorders worldwide is Attention deficit hyperactivity disorder (ADHD)^1^. The disorder is characterized by cardinal symptoms of age-inappropriate inattention (IA), hyperactivity (HA), and impulsivity (Imp)^1^. High heritability, genetic association studies, and case–control linkage analyses indicate a significant role of genetics in the etiology^2–4^. In addition, environmental factors like low birth weight, prenatal exposure to nicotine, alcohol, drugs, and adverse life experiences have also shown significant contributions to ADHD^3,4^.

Although molecular genetic studies were primarily focused on catecholaminergic dysregulation, the glutamatergic system was also reported to influence ADHD aetiology^5^. Functional abnormalities of the prefrontal cortex (PFC), a brain region regulating executive functions including working memory, sustained attention, decision making, and emotional control, were documented in subjects with ADHD^6^. The glutamatergic pyramidal neurons are the PFC's major cellular constituents; hence, a primary role of glutamatergic neurotransmission in PFC-dependent executive functions was speculated^7,8^. The influence of glutamate in ADHD was also evident from the hyperfunctional glutamatergic system in the PFC of spontaneously hypertensive rats^9^. Methylphenidate (MPH), a psychostimulant used for treating subjects with ADHD^10^, was reported to target the glutamate receptors in the PFC neurons^11–13^.

The action of glutamate, the most abundant excitatory neurotransmitter, is mediated through the glutamate receptors (GluRs) located chiefly on the membranes of the neuronal and glial cells^14^. The GluR, responsible for post-synaptic excitation of neural cells, are subdivided into metabotropic (mGluR) and ionotropic (iGluR) receptors based on their pharmacological properties^15^. The mGluR, capable of increasing or decreasing the excitability of the post-synaptic cells, is a class C family of G-protein coupled receptors including eight members, mGluR1/GRM1-mGluR8/GRM8) and induces a slow response through a signal transduction cascade^16^. On the other hand, the ionotropic receptors (iGluR) mediate fast excitatory neurotransmission through three ligand-gated ion channels, N-methyl-D-aspartate (NMDA), α-amino-3-hydroxy-5-methyl-4-isoxazole propionic acid (AMPA), and kainate receptors (KARs)^17,18^.

The kainate receptors (KAR), encoded by *GRIK1–GRIK5* genes, form functional ion channels by combinations of five different subunits, i.e., GluK1–GluK5^17,19^. KARs modulate both glutamate and GABA release^20–23^. Hippocampal KARs containing GluK1 subunits are primarily expressed in interneurons, where the reduction of GABA release results in the increased excitability of glutamatergic principal neurons^23^. In the pyramidal cells of the hippocampus, KARs activation leads to G-protein activation and phospholipase C and protein kinase C signaling, which decreases GABA release^20^. On the other hand, in the hippocampal mossy fiber-CA3 synapses, medial geniculate nucleus at the thalamus, and lateral amygdala synapses, metabotropic action of KARs leads to a reduction in glutamate release resulting in long-term depression^21,22^. Glutamate release in the Schaffer collateral-CA2 synapses of the hippocampus^24^ and cerebellum synapses^25^ is also affected by KARs, which involve G-protein and protein kinase A activation. The metabotropic action of KARs also takes place in a biphasic manner. At the same time, the G-protein independent, Ca^2+^ dependent intracellular signaling cascade facilitates glutamate release, G-protein and Ca^2+^ dependent signaling cascade decrease the glutamate release^23,26,27^. Further, the pre-synaptic KARs activation through Ca^2+^—calmodulin complex was also reported to stimulate glutamate release in the hippocampal mossy fiber-CA3 synapses, thalamocortical synapses, and cerebrocortical synaptosomes, thereby inducing the long-term potentiation^26,28,29^.

Several investigations were carried out to determine the role of KARs in disorders such as epilepsy, pain, ischemic brain injury, stress and anxiety, ASD, schizophrenia, alcohol abuse disorder, bipolar disorder, and depression^30–32^. Irregular KARs activity in the temporal lobe was reported to induce epileptogenic neuronal activity at the hippocampal synapses^33^. The GluK2 subunit was found to have a role in seizure^34,35^, and a bi-allelic loss of function mutation in the KARS-encoding gene, *GRIK2*, was detected in patients with neurodevelopmental disorders^36^. Abnormalities in the kainate receptors were also reported in patients with bipolar disorder, autism, and intellectual disability^37^. Genome-wide association studies identified a *GRIK1* intronic variant as a candidate for ADHD^38^. However, no exploration was performed on the association between *GRIK1* exonic variants and ADHD traits. We, for the first time, investigated two *GRIK1* exonic variants, rs363504 [c.2705T > C (p.Leu902Ser)] and rs363538 [c.522A/C (p.Thr)], in a group of ethnically matched subjects by case–control as well as family-based methods to identify their association with ADHD, different traits, post-therapeutic changes in the trait scores of the ADHD probands and GRIK1 expression in the peripheral blood.

## Results

The *GRIK1* genetic variants were analyzed in a group of ethnically matched families with ADHD probands and controls. Genotyping success rates were 99% and 98% for rs363504 and rs363538, respectively. Genotypic frequencies followed the Hardy–Weinberg equilibrium (*P* > 0.05) in all the case and control groups.

### Population-based analysis

Population-based comparative analysis revealed an absence of the rs363504 ‘CC’ genotype in the control group (Table 1), while this genotype was detected in the probands (*P* = 0.04, OR = 9.74, CI = 1.00–95.45; Power 40%). The probands also showed a higher frequency of the rs363538 ‘CA’ genotype (*P* = 0.05; OR = 1.50, CI = 0.97–3.00; Power 39%).

**Table 1:** Allelic and genotypic frequencies of GRIK1 variants in the studied population.

rsID	Allele/Genotype	Allelic/genotypic frequencies (Count)								
		Control	Probands	χ^2^ (P)	Male control	Father	χ^2^ (P)	Female control	Mother	χ^2^ (P)
rs363504	T	0.94 (647)	0.92 (495)	1.42 (0.23)	0.93 (280)	0.92 (352)	1.63 (0.20)	0.94 (367)	0.95 (423)	0.20 (0.65)
	C	0.06 (43)	0.08 (44)		0.07 (22)	0.08 (32)		0.05 (21)	0.05 (21)	
	TT	0.88 (302)	0.85 (229)	0.74 (0.38)	0.85 (129)	0.84 (162)	1.04 (0.30)	0.89 (173)	0.91 (201)	0.21 (0.64)
	TC	0.12 (43)	0.14 (37)	0.22 (0.63)	0.15 (22)	0.15 (28)	0.48 (0.48)	0.11 (21)	0.09 (21)	
	CC	0 (0)	0.01 (3)	**3.87 (0.04)**	0 (0)	0.01 (2)	**3.60 (0.05)**	–	–	
rs363538	C	0.14 (97)	0.17 (93)	2.17 (0.14)	0.14 (43)	0.20 (75)	**4.83 (0.02)**	0.14 (54)	0.18 (78)	2.14 (0.14)
	A	0.86 (581)	0.83 (441)		0.86 (255)	0.80 (309)		0.86 (326)	0.82 (366)	
	CC	0.02 (7)	0.02 (5)	0.03 (0.86)	0.01 (2)	0.03 (6)	1.16 (0.28)	0.03 (5)	0.02 (4)	0.33 (0.56)
	CA	0.25 (83)	0.31 (83)	**3.72 (0.05)**	0.26 (39)	0.33 (63)	**4.26 (0.03)**	0.23 (44)	0.32 (70)	**3.58 (0.05)**
	AA	0.73 (249)	0.67 (179)	3.16 (0.07)	0.72 (108)	0.64 (123)	2.72 (0.09)	0.74 (141)	0.66 (148)	2.78 (0.09)

N.B: Statistically significant differences are presented in bold.

The gender-based stratified analysis on allele/genotype frequencies failed to show significant differences between the probands and the controls (*P* > 0.09; Supplementary Table S1).

The father of the probands showed a higher occurrence of the rs363504 ‘CC’ genotype (Table 1; *P* = 0.05; OR = 1.32, CI = 0.65–2.12; Power 38%), rs363538 ‘C’ allele (*P* = 0.02; OR = 1.46, CI = 1.04–2.06; Power 59%) and ‘CA’ genotype (*P* = 0.03; OR = 1.53, CI = 1.04–2.28; Power 44%). The mother showed a higher frequency of rs363538 ‘CA’ genotype (*P* = 0.05; OR = 1.54, CI = 1.03–2.28; Power 38%) than the gender-matched controls.

### Family-based transmission analysis

The transmission disequilibrium test (TDT) revealed biased maternal transmission of the rs363504 ‘C’ allele (*P* = 0.05; RR = 0.47, CI = 0.26–0.86; Power 47%) to the probands, chiefly to the male probands (*P* = 0.03; RR = 0.40, CI = 0.20–0.77; Power 58%) (Table 2). Biased transmission of the rs363538 ‘A’ allele was also observed in the female probands (*P* = 0.05; RR = 0.32, CI = 0.09–1.21; Power 46%) from both the parents as well as the mother (*P* = 0.05; RR = 0.37, CI = 0.14–0.98; Power 46%) (Table 2).

**Table 2:** Analysis of parental transmission to the probands.

Variant	Parent	Proband	Allele	Frequency (Count)		χ^2^ (P)
				T	NT	
rs363504	Mother	Both	T	0.94 (452)	0.97 (468)	3.54 (0.05)
			C	0.06 (30)	0.03 (14)	
	Mother	Male	T	0.94 (406)	0.98 (423)	4.69 (0.03)
			C	0.06 (26)	0.02 (10)	
rs363538	Both	Female	C	0.12 (3)	0.31 (8)	3.50 (0.05)
			A	0.88 (23)	0.69 (18)	
	Mother	Female	C	0.12 (6)	0.29 (14)	3.48 (0.05)
			A	0.88 (42)	0.71 (34)	

N.B: T = Transmitted; NT = Not Transmitted.

### Quantitative trait analysis

Genotype–phenotype association analysis (Table 3) revealed a positive influence of the rs363504 ‘T’ allele (*P* = 0.05, Add Value = 0.01) and ‘TT’ genotype (*P* = 0.04, Add Value = 0.01) on the hyperactivity (HA) score measured using the Conners Parent and Teacher Rating Scale-Revised (CPRS-R). No significant association was detected for other traits assessed by the CPRS-R (Supplementary Table S2). rs363504 ‘T’ allele also showed positive influence (Table 3) on the oppositional defiant disorder (ODD; Add Value = 0.06; *P* = 0.005) and Parental Account of Children’s Symptoms (PACS; Add Value = 0.10; *P* = 0.003) scores. Probands with genotypes having the “T” alleles (Table 3) also showed higher scores for ODD (*P* < 0.02, AddValue = 12.6) and PACS (*P* < 0.007, Add Value = 16.03).

**Table 3:** Quantitative trait analysis involving gene variants and ADHD-associated traits.

Variant	Trait	Allele/Genotype/Haplotype	Add value	χ^2^ (P)	CI
rs363504	HA (CPRS)	T	0.01	**3.65 (0.05)**	−0.0005 to 0.02
		C	−0.01		−0.02 to 0.0005
		TT	0.01	**3.2 (0.04)**	−0.01 to 0.04
		TC	−0.006	0.83 (0.16)	−0.02 to 0.03
		CC	−0.01	0.46 (0.50)	−0.04 to 0.02
	ODD	T	0.06	**7.68 (0.005)**	0.01 to 0.10
		C	−0.06		−0.10 to 0.01
		TT	12.6	**6.80 (0.009)**	−118.5 to 143.6
		TC	12.5	**5.28 (0.02)**	−118.6 to 143.6
		CC	−27.09	1.65 (0.19)	−27.09 to 27.09
	PACS	T	0.10	**8.90 (0.003)**	0.03 to 0.17
		C	−0.10		−0.17 to 0.02
		TT	16.12	**8.48 (0.004)**	−52.14 to 84.39
		TC	16.03	**7.24 (0.007)**	−52.14 to 84.39
		CC	−10.97	1.06 (0.30)	−10.97 to 10.97
rs363538	HI (DSM)	C	−0.05	**3.95 (0.04)**	−0.09 to 0.002
		A	0.05		0.002 to 0.09
		CC	0.01	0.32 (0.57)	−0.07 to 0.10
		CA	−0.08	**7.02 (0.008)**	−0.13 to 0.02
		AA	0.07	**5.99 (0.01)**	0.02 to 0.01
	IQ	C	0.28	1.15 (0.28)	−0.80 to 0.23
		A	−0.28		−0.23 to 0.80
		CC	19.67	**3.58 (0.05)**	19.21 to 20.13
		CA	0.64	**4.92 (0.02)**	−1.25 to 0.03
		AA	−0.52	2.92 (0.08)	−0.07 to 1.12
rs363504–rs363538	HI (DSM)	C-A	0.02	2.82 (0.09)	−0.006 to 0.06
		C–C	−0.01	0.02 (0.87)	−0.11 to 0.09
		T–A	−0.02	0.75 (0.38)	−0.05 to 0.01
		T–C	−0.07	**4.57 (0.03)**	−0.02 to 0.01
	ODD	C–A	−0.08	**7.68 (0.005)**	−0.14 to 0.02
		C–C	−13.53	2.18 (0.13)	−13.53 to 13.53
		T–A	0.05	1.66 (0.19)	0.003 to 0.10
		T–C	0.06	0.47 (0.48)	0.007 to 0.11
	PACS	C–A	−0.10	**6.54 (0.01)**	−0.17 to 0.01
		C–C	−17.08	2.70 (0.10)	−17.08 to 17.08
		T–A	0.08	1.70 (0.19)	0.009 to 0.15
		T–C	0.09	0.44 (0.50)	0.01 to 0.16

N.B: Statistically significant differences are presented in bold. HA(CPRS)- Hyperactivity score (Conner’s Parent Rating Scale-Revised); ODD- Oppositional Defiant disorder, PACS- Parental Account of Children’s Symptoms; HI(DSM)- Hyperactivity/Impulsivity (Diagnostic and Statistical Manual); IQ- Intelligence Quotient.

Scores for Diagnostic and Statistical Manual-Hyperactivity/Impulsivity (DSM-HI) were higher in the presence of the rs363538 ‘A’ allele (*P* = 0.04, Add Value = 0.05) and ‘AA’ genotype (*P* = 0.01, Add Value = 0.07), while the traits scores (*P* = 0.008, Add Value = −0.08) were reduced in the presence of the ‘CA’ genotype (Table 3). The other traits, i.e., inattention (IA) ADHD index (AI), and behavioral problem (BPr), assessed through DSM, CPRS-R, ODD, and PACS, respectively, did not show any significant influence on the studied variants (Supplementary Table S2). Probands harbouring the ‘CC’ (*P* = 0.05, Add Value = 19.67) and ‘CA’ (*P* = 0.02, Add Value = 0.64) genotypes showed higher IQ scores (Table 3). The presence of the ‘T-C’ haplotype, formed by rs363504–rs363538, lowered the score for DSM-HI (*P* = 0.03), whereas the ‘C-A’ haplotype had a negative impact on ODD (*P* = 0.005) and PACS (*P* = 0.01) scores (Table 3).

### Association of the genetic variants with pharmacotherapy

**Figure 1:**
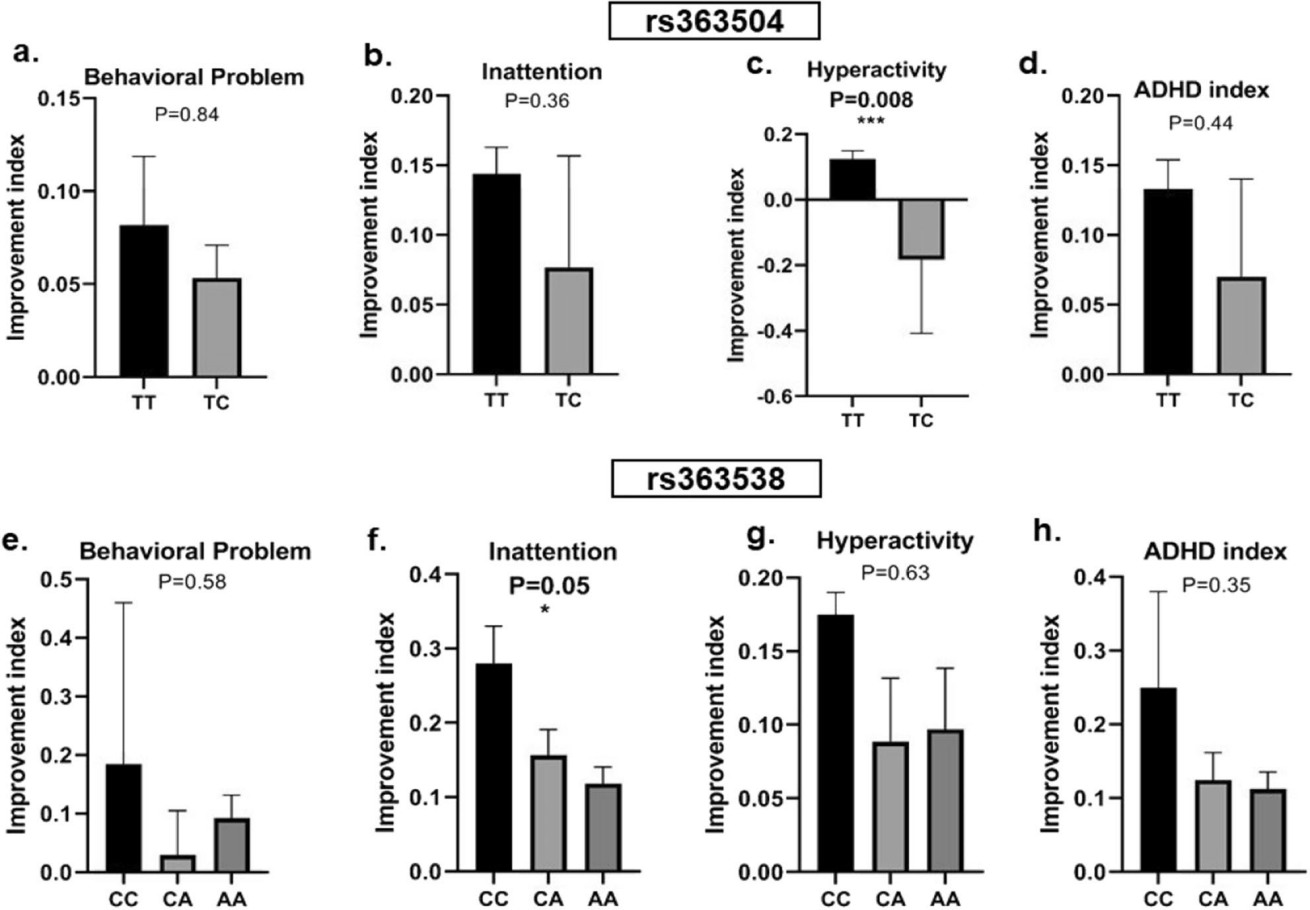
Association between GRIK1 genetic variants and methylphenidate-induced changes in the trait scores, measured based on the improvement index, was analyzed by the Unpaired t-test using the Prism 9.0 software; (**a**, **e**) Behavioral problem; (**b**, **f**) Inattention; (**c**, **g**) Hyperactivity; (**d**, **h**) ADHD index. Statistically, significant differences are presented in bold (*).

The ADHD probands were treated with either stimulant (MPH) or non-stimulant (atomoxetine/ATX) medications based on the age at presentation, presenting symptoms, and availability of the medicine, and the treatment efficacy was tested in the probands having different *GRIK1* genotypes. Probands carrying the rs363504 ‘TT’ genotype showed significant improvement in the scores for HA after MPH treatment (Fig. 1c). Marginally higher scores for BPr, IA, and AI were also observed in the presence of the ‘TT’ genotype (Fig. 1a,b,d). Probands harboring rs363538 ‘CC’ genotype showed a trend for improvement in the trait scores (Fig. 1e–h) with a significant impact on the IA score (Fig. 1f). Treatment with ATX failed to show any statistically significant association with the studied genetic variants (Supplementary Fig. 1).

### Analysis of GRIK1 mRNA expression

**Figure 2:**
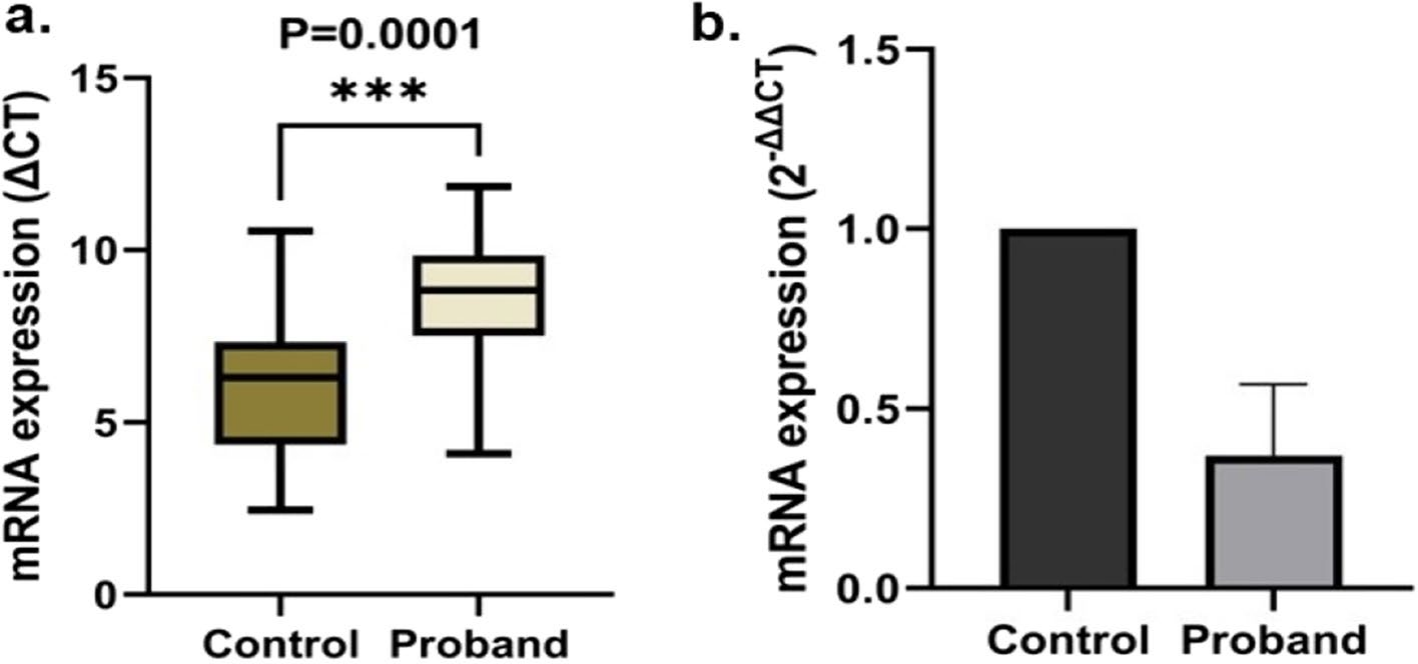
Relative GRIK1 mRNA expression in the peripheral blood of controls and ADHD probands were analyzed by the Unpaired t-test using the Prism 9.0 software; a. box plot shows ΔCT values, b. bar diagram shows the fold change in expression.

**S**tatistically significant lower expression (t = 4.40, *P* = 0.0001) of GRIK1 mRNA was detected in the peripheral blood of the ADHD probands as compared to the age-matched control (Control Mean ΔCT = 5.90 ± 0.46; Proband Mean ΔCT = 8.67 ± 0.42) (Fig. 2a). In addition, the comparative analysis showed 2.70-fold downregulation in the expression of GRIK1 in the ADHD probands (Fig. 2b).

## Discussion

We investigated the association of two functional *GRIK1* variants, rs363504 and rs363538, with ADHD. The data obtained indicated that these variants influence the phenotypic attributes of the ADHD probands before and after therapeutic intervention. Biased maternal transmission of the rs363504 ‘C’ and rs363538 ‘A’ alleles to the probands was also detected. GRIK1 expression was found to be significantly downregulated in the peripheral blood of the probands.

KARs have major modulatory roles in both glutamate and GABA release^20–22^. Pre-synaptic KARs exerts a biphasic effect on the release of neurotransmitters; low doses of agonists increase the release of neurotransmitters, while higher concentrations produce a decrease in evoked excitatory post-synaptic currents^27,32,39^. However, though the effect of KARs, mediated through the protein kinase C pathway, is well documented^27,32,39^, investigators have also observed that KARs activities are not adequately ionotropic or mediated by protein kinases^40^. It was also found that protein kinase A-mediated regulation is not restricted to the hippocampus or the amygdala but extends to the cerebellum^25^. Hence, we wanted to determine whether KARs have any role in ADHD and analyzed functional GRIK1 variants in a group of ethnically matched subjects, including ADHD probands.

GRIK1 containing glutamate receptors are expressed in the brain regions^41^ necessary for learning and memory^42^. The *GRIK1* gene at chromosome 21q22.1 spans 402 kb and is divided into 18 exons. In addition, genome-wide association studies suggested that *GRIK1*^38^ and *GRIK4*^43^ might be associated with the risk of ADHD. However, no significant association was reported between ADHD and the *GRIK1* exonic variants, ADHD traits, pre-and post-medication changes in ADHD phenotypes, and GRIK1 mRNA expression in the peripheral blood.

rs363504 is a T to C transition at codon 902 (T2705C) in exon 17 and results in a non-synonymous change (Leu902Ser) that affects the intracellular C-terminal domain of the receptor subunit. In the European and African populations, the rs363504 ‘TT’ genotype showed an association with opioid and cocaine dependency^44^. This gene variant also exhibited a potential contribution to the severity of schizophrenia and ASD^37^ in the European subjects, while no significant association was found in the Japanese patients with schizophrenia^45^. Our study on the Indo-Caucasoid subjects revealed an absence of the rs363504 ‘CC’ genotype in the control subjects, while this genotype was detected in the ADHD probands and their fathers. Family-based analysis revealed biased maternal transmission of the ‘C’ allele to the ADHD probands, chiefly to the male probands. Based on these data, we may postulate that the rs363504 ‘C’ has a role in the etiology of ADHD, at least in this population. On the other hand, the genotype–phenotype association analysis revealed higher scores for HA, ODD, and PACS in the presence of the rs363504 ‘T’ allele or ‘TT’ genotype. Since the “T” allele is the ancestral and major allele, a further functional investigation is warranted to determine the role of this variant/s in the disease etiology.

rs363538 (C522A) is localized in the exon 3, causing a silent transversion coding for Thr^174^ and affecting the extracellular ligand-binding loop^46,47^. Population-based analysis revealed a higher frequency of the rs363538 ‘CA’ genotype in the probands with a concomitantly lower frequency of the “AA” genotype. Familial transmission analysis showed higher parental transmission of the rs363538 'A' allele to the female probands, chiefly due to higher maternal transmission. QTA revealed a higher IA score in the presence of rs363538 ‘A’/’AA’, whereas the probands harboring the ‘CA’ genotype showed lower IA scores predominantly due to the ‘C’ allele. The IQ score was higher in the presence of the ‘C’ allele. The probands carrying the ‘CC’ genotype on MPH treatment showed improved IA scores. Based on these data, we conclude that the probands with rs363538 ‘C’ allele may have a better function of the GRIK1, and those with the “A” allele, especially the female probands, require more intensive intervention due to low IQ and higher IA.

GRIK1 mRNA expression was 2.70-fold downregulated in the ADHD probands. Analysis of correlation using mean values of normalized mRNA levels revealed that the ADHD probands having the rs363504 ‘TT (t = 0.25, *P* = 0.53) and rs363538 ‘AA’ (t = 1.78, *P* = 0.08) genotypes had lower GRIK1 expression (Data not presented due to limitation in the number of samples after stratified analysis). However, the same genotypes, i.e., rs363504 ‘TT’ and rs363538 ‘AA’, were found to increase the trait scores. In experimental rodents, a behavioral abnormality was induced by altered GABAergic transmission due to loss of modulation of GRIK1 in the amygdala neurons^48^. Similar downregulation in the GRIK1 expression in the peripheral blood of the ADHD probands may indicate an alteration in GABAergic transmission. Based on this information, it can be concluded that GRIK1 may be a potential candidate for ADHD, which requires further in-depth investigation.

In experimental animal models of ADHD, administration of MPH was reported to decrease behavioral deficits while improving the glutamatergic signaling mediated through ionotropic glutamate receptors^11–13^. Our investigation showed that the probands carrying the rs363504 ‘TT’ genotype significantly improved the HA scores after MPH treatment. BPr, IA, and AI scores were also lowered after MPH treatment. On the other hand, probands with rs363538 ‘AA’ genotype exhibited a lack of significant improvement in the trait scores. In contrast, those with the ‘CC’ genotype showed improvement in all the trait scores, the most significant being IA. The rs363504 “T” and rs363538 ‘C’ are the ancestral alleles that may encode for a GRIK1 receptor with normal function. Therefore, MPH-induced improvement in the trait scores in the presence of rs363504 “T” and rs363538 ‘C’ alleles indicate that normal functioning of GRIK1 is required to achieve successful remediation of ADHD traits after MPH treatment.

ATX was reported to alter the electrophysiological activity of the PFC neurons through modulation of the NMDAR-mediated glutamatergic transmission^49,50^. We speculated that ATX might also affect the KARs mediated synaptic transmission since the metabotropic function of KAR initiates G protein activation^23^ and G-protein-activated inwardly rectifying K + channels were reported to be inhibited by ATX^51^. Our investigation of *GRIK1* genetic variants showed a very low frequency of rs363538 “CC” genotypes in both the controls and the probands. The ADHD probands having the rs363504 “TT” and rs363538 “CA” genotypes showed improvement in the IA, HA, and AI scores after ATX treatment. This trend for ATX-induced improvement in the presence of the rs363504 and rs363538 ancestral alleles could be mediated, at least partially, by modulation of the glutamatergic function. However, this improvement in the trait scores after ATX treatment was not statistically significant compared to the improvement observed after MPH treatment. A recent comparative analysis of 456 children and adolescents with ADHD also showed better outcomes following MPH treatment than ATX^52^.

For the first time, this study showed significant associations between *GRIK1* genetic variants with (1) ADHD-associated traits and (2) MPH-induced changes in the trait scores of the ADHD probands. In addition, we have detected significant downregulation in GRIK1 mRNA expression in the probands. The primary limitations of the present study are (a) the analysis of GRIK1 mRNA expression in only a limited number of samples and (b) marginally significant associations between the studied genetic variants and ADHD, as is evident from the close to significance p-values, low relative risk, and power of the association tests. However, based on the significant associations of the studied genetic variants with ADHD traits, improvement in the trait scores after MPH treatment, and down-regulation in GRIK1 expression, a possible role of GRIK1 can be predicted in the etiology of ADHD, which warrants further validation in a large cohort of subjects belonging to different ethnicities.

## Materials and methods

### Recruitments of subjects and assessment of traits

Nuclear families with ADHD probands (N = 272; mean age 8.80 ± 3.46; male: female ratio 10:1) were recruited following the DSM^1,53^. Behavioral problems (BPr), inattention/cognitive problems (IA), hyperactivity (HA), and ADHD index (AI) were assessed using the Conner’s Parents Rating Scale-Revised (CPRS-R)^54^. Parental Account of Children’s Symptoms (PACS) was used to evaluate the severity of BPr^55^. Scores for the co-morbid oppositional defiant disorder (ODD) were assessed based on the DSM criteria. Intelligence Quotient (IQ) was determined using the Wechsler’s Intelligence Scale for Children-III^56^. Ethnically matched control subjects (N = 352; mean age 8.9 ± 6.8; male: female ratio 10:3) were also recruited following the DSM criteria. Written informed consent was obtained from the participants/ parents/caregivers during recruitment. All methods were performed per the relevant guidelines and regulations. The study protocol (No. PR-003-17) was approved by the Manovikas Ethics Committee on Human Subjects, having Scientists, Psychiatrists, Psychologists, Advocates, and Social workers as members.

### Sample collection and genotyping of the target sites

At the time of recruitment, peripheral blood was collected from treatment naïve ADHD probands, their parents, and controls. Genomic DNA was isolated by the phenol/chloroform method^57^. Genotyping of rs363504 and rs363538 was performed by polymerase chain reaction (PCR) amplification in the Applied Biosystems ProFlex™ PCR system, followed by Restriction fragment length polymorphism analysis using Ase I and Btg I enzymes, respectively.

### Pharmaceutical intervention

Methylphenidate at a dose of 0.3 mg/kg body weight/day was prescribed for two months to ADHD probands with age-inappropriate HA, residing in urban areas and < 10 years of age, followed by 0.6 mg/kg body weight/day for another four months. ADHD probands with significant IA, > 10 years of age, and residing in rural areas where availability of stimulant medication is limited, were prescribed ATX at a dose of 0.8 mg/kg body weight/ day for two months, followed by 1.2 mg/kg body weight/day for another four months. Probands available for follow-up after six months of treatment (N = 74) were re-assessed by the CPRS-R.

### Analysis of GRIK1 mRNA expression

RNA was isolated from the blood by the TRIzol method (TRIzol Reagent User Guide; Pub.No. MAN0001271 B.0), and following DNAase treatment, the concentration of isolated RNA was measured by a Qubit 4 Fluorometer. The total RNA (700 ng) was reverse transcribed into complementary DNA (cDNA) using the reverse transcriptase enzyme (High-capacity cDNA reverse transcription kit of Thermo Fischer Scientific). GRIK1 mRNA expressions were examined in the ADHD probands (n = 17) and age-matched control subjects (n = 19). Amplification was carried out in QuantStudio 3 (Applied Biosystems by Thermo Fisher Scientific) using PowerUp SYBR Green Master Mix (Thermo Fisher Scientific). The cycle threshold value (Ct value) for each sample was obtained. The data was normalized against Glyceraldehyde 3-phosphate dehydrogenase (GAPDH) expression, serving as an internal control, and expressed as ΔCt. Normalized gene expression or fold change is defined as 2^−ΔΔCt^.

### Statistical analysis

Hardy Weinberg Equilibrium (HWE) was calculated using online software (http://www.oege.org/software/hwe-mr-calc.shtml/) to determine the genotypic frequencies of the studied variants were constant or not. Population-based comparative analysis and family-based transmission analyses were performed using the UNPHASED version 3.1.7^58^, after 1000 permutations, which takes care of the multiple corrections. Quantitative trait (QT) analysis to identify the association between the genetic variants and ADHD-associated trait scores was also performed using the UNPHASED version 3.1.7^58^. Odds ratio (OR) and confidence intervals (CI) were calculated using the Odds Ratio calculator (http://www.hutchon.net/ConfidORnulhypo.htm). Relative Risk (RR) of the studied variants was calculated using a relative risk calculator, and the Power of the significant observations was calculated using piface software^59^. Relative GRIK1 mRNA expression in the peripheral blood of controls and ADHD probands were analyzed by the Unpaired t-test using Prism 9.0 (GraphPad Software, Inc). Improvement in the trait scores after pharmaceutical interventions were calculated by 1-Tn/To (To = initial trait score, Tn = Post-treatment trait score) as detailed in a previous article^60^ and are presented as the improvement index. Association between *GRIK1* variants and treatment-induced changes in the trait scores, measured based on the improvement index, was analyzed by the Unpaired t-test using the Prism 9.0 software, and data from the unpaired t-test are presented as Mean ± Standard error of the mean (SEM).

## Supplementary Information

Supplementary Information 1.

Supplementary Information 2.

## Data Availability

The datasets used and analyzed during the current study are available from the corresponding author upon reasonable request.
