# Supplementary material for: Kainate receptor subunit 1 (GRIK1) risk variants and GRIK1 deficiency were detected in the Indian ADHD probands
Source: Sci Rep. 2022 Nov 2;12:18449. doi: 10.1038/s41598-022-21948-0 (PMC9630447; doi:10.1038/s41598-022-21948-0)

Supplementary Figure 1: Association between genetic variants and changes in the trait scores after atomoxetine treatment.

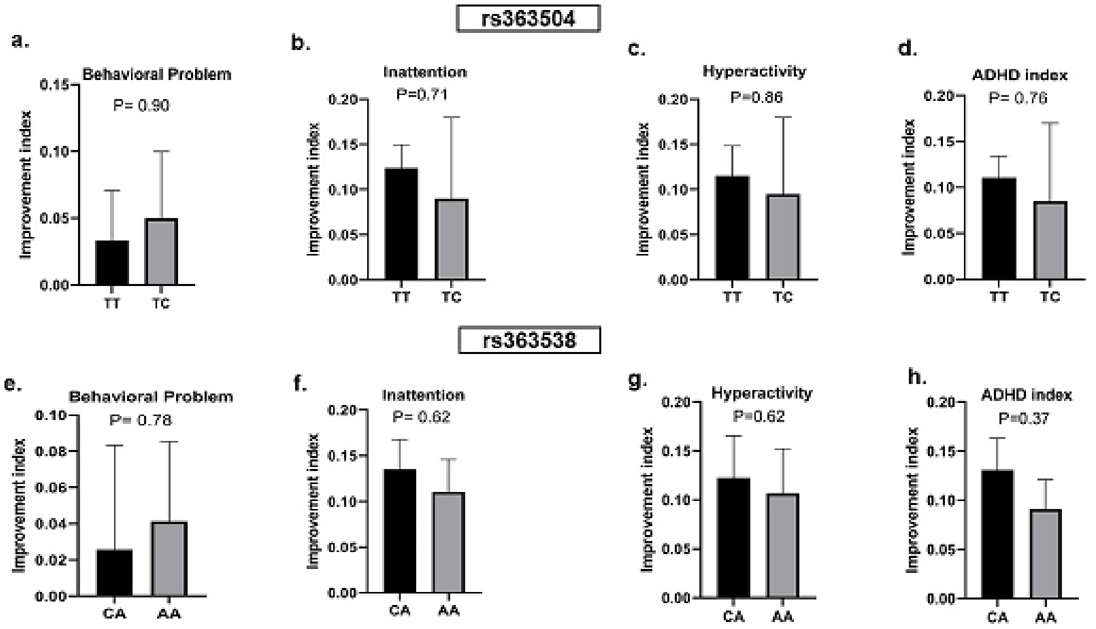

Supplementary Figure 2: GRIK1 mRNA expression in the ADHD probands in the presence of the genetic variants.

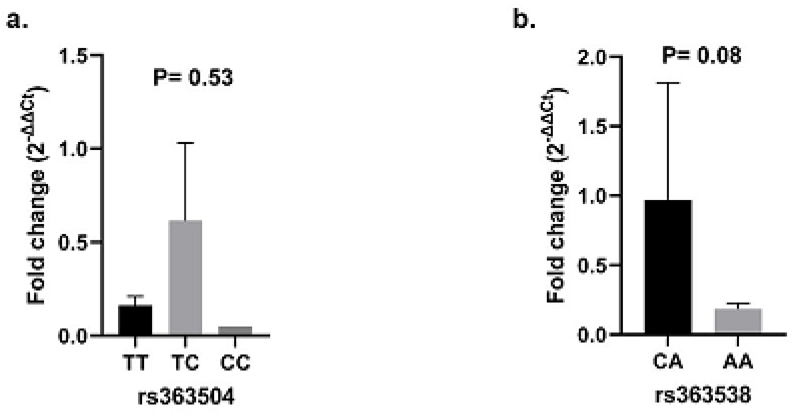

Supplement: Supplementary file 1 — Supplementary Information 1. [file 41598_2022_21948_MOESM1_ESM.pdf]
