# Supplementary material for: Kainate receptor subunit 1 (GRIK1) risk variants and GRIK1 deficiency were detected in the Indian ADHD probands
Source: Sci Rep. 2022 Nov 2;12:18449. doi: 10.1038/s41598-022-21948-0 (PMC9630447; doi:10.1038/s41598-022-21948-0)
Supplement: Supplementary file 2 — Supplementary Information 2. [file 41598_2022_21948_MOESM2_ESM.docx]

Table S1: Gender-based stratified analysis on allelic and genotypic frequencies

| Variant | Allele/  Genotype | Male control | Male proband | χ2(P) | Female control | Female proband | χ2(P) |
| --- | --- | --- | --- | --- | --- | --- | --- |
| rs363504 | T | 0.93 | 0.92 | 0.06 (0.80) | 0.95 | 0.90 | 1.72 (0.18) |
|  | C | 0.07 | 0.08 |  | 0.05 | 0.10 |  |
|  | TT | 0.85 | 0.853 | 2.00 (0.36) | 0.89 | 0.84 | 4.15 (0.09) |
|  | TC | 0.15 | 0.139 |  | 0.11 | 0.13 |  |
|  | CC | 0 | 0.008 |  | 0 | 0.03 |  |
| rs363538 | C | 0.14 | 0.18 | 1.84 (0.17) | 0.14 | 0.12 | 0.29 (0.58) |
|  | A | 0.86 | 0.82 |  | 0.86 | 0.88 |  |
|  | CC | 0.01 | 0.02 | 1.98 (0.37) | 0.03 | 0 | 1.48 (0.47) |
|  | CA | 0.26 | 0.32 |  | 0.23 | 0.23 |  |
|  | AA | 0.73 | 0.66 |  | 0.74 | 0.77 |  |

Table S2: Quantitative trait analysis to identify the association between genetic variants and traits.

| Variant | Trait | Allele/Genotype/ Haplotype | Add Value | χ2 (P) | CI |
| --- | --- | --- | --- | --- | --- |
| rs363504 | IA (DSM) | T | 0.0002 | 0.002 (0.97) | -0.01 to 0.01 |
|  |  | C | -0.0002 |  | -0.01 to 0.01 |
|  |  | TT | 0.00007 | 0.001 (0.99) | -0.03 to 0.03 |
|  |  | TC | -0.0002 |  | -0.04 to 0.04 |
|  |  | CC | -0.00003 |  | -0.03 to 0.03 |
|  | HI (DSM) | T | -0.02 | 1.78 (0.18) | -0.05 to 0.008 |
|  |  | C | 0.02 |  | -0.008 to 0.06 |
|  |  | TT | -0.04 | 1.64 (0.43) | -0.12 to 0.03 |
|  |  | TC | -0.02 |  | -0.09 to 0.06 |
|  |  | CC | 0.03 |  | -0.03 to 0.11 |
|  | BPr (CPRS) | T | -0.0007 | 0.01 (0.89) | -0.01 to 0.01 |
|  |  | C | 0.0007 |  | -0.01 to 0.01 |
|  |  | TT | 0.007 | 0.29 (0.86) | -0.03 to 0.04 |
|  |  | TC | 0.009 |  | -0.02 to 0.05 |
|  |  | CC | -0.007 |  | -0.04 to 0.03 |
|  | IA (CPRS) | T | 0.0002 | 0.001 (0.96) | -0.01 to 0.01 |
|  |  | C | -0.0002 |  | -0.01 to 0.01 |
|  |  | TT | 0.0007 | 0.001 (0.99) | -0.04 to 0.04 |
|  |  | TC | -0.0001 |  | -0.04 to 0.04 |
|  |  | CC | -0.0003 |  | -0.04 to 0.04 |
|  | AI (CPRS) | T | 0.003 | 0.21 (0.64) | -0.008 to 0.01 |
|  |  | C | -0.003 |  | -0.01 to 0.008 |
|  |  | TT | 0.008 | 0.02 (0.65) | -0.03 to 0.04 |
|  |  | TC | 0.007 |  | -0.03 to 0.04 |
|  |  | CC | -0.008 |  | -0.04 to 0.03 |
|  | IQ | T | -0.53 | 1.57 (0.21) | -1.37 to 0.31 |
|  |  | C | 0.53 |  | -0.30 to 1.36 |
|  |  | TT | -0.23 | 1.78 (0.40) | -3.01 to 2.56 |
|  |  | TC | 0.40 |  | -2.50 to 3.31 |
|  |  | CC | 0.16 |  | -2.62 to 2.95 |
| rs363538 | IA (DSM) | C | 0.001 | 0.09 (0.76) | -0.007 to 0.009 |
|  |  | A | -0.001 |  | -0.008 to 0.007 |
|  |  | CC | 0.02 | 1.30 (0.52) | -0.02 to 0.08 |
|  |  | CA | -0.0006 |  | -0.01 to 0.008 |
|  |  | AA | -0.0002 |  | -0.009 to 0.009 |
|  | BPr (CPRS) | C | 0.003 | 0.42 (0.52) | -0.005 to 0.01 |
|  |  | A | -0.003 |  | -0.01 to 0.005 |
|  |  | CC | 0.04 | 2.97 (0.22) | -0.01 to 0.10 |
|  |  | CA | 0.0001 |  | -0.009 to 0.009 |
|  |  | AA | -0.001 |  | -0.01 to 0.008 |
|  | IA (CPRS) | C | 0.001 | 0.09 (0.76) | -0.007 to 0.009 |
|  |  | A | -0.001 |  | -0.009 to 0.007 |
|  |  | CC | 0.02 | 0.003 (0.95) | -0.02 to 0.07 |
|  |  | CA | -0.0006 |  | -0.009 to 0.008 |
|  |  | AA | -0.0002 |  | -0.009 to 0.009 |
|  | HA (CPRS) | C | -0.001 | 0.15 (0.69) | -0.008 to 0.005 |
|  |  | A | 0.001 |  | -0.005 to 0.008 |
|  |  | CC | 0.009 | 0.93 (0.62) | -0.02 to 0.05 |
|  |  | CA | -0.003 |  | -0.01 to 0.005 |
|  |  | AA | 0.002 |  | -0.005 to 0.01 |
|  | AI (CPRS) | C | 0.002 | 0.29 (0.58) | -0.005 to 0.01 |
|  |  | A | -0.002 |  | -0.01 to 0.005 |
|  |  | CC | 0.03 | 1.66 (0.43) | -0.03 to 0.10 |
|  |  | CA | 0.0004 |  | -0.008 to 0.01 |
|  |  | AA | -0.001 |  | -0.01 to 0.007 |
|  | ODD | C | 0.003 | 0.06 (0.79) | -0.01 to 0.02 |
|  |  | A | -0.003 |  | -0.02 to 0.02 |
|  |  | CC | 0.02 | 0.32 (0.85) | -0.05 to 0.10 |
|  |  | CA | 0.0008 |  | -0.02 to 0.03 |
|  |  | AA | -0.002 |  | -0.02 to 0.02 |
|  | PACS | C | 0.001 | 0.01 (0.89) | -0.02 to 0.02 |
|  |  | A | -0.001 |  | -0.02 to 0.02 |
|  |  | CC | 0.01 | 0.09 (0.95) | -0.06 to 0.09 |
|  |  | CA | -0.0009 |  | -0.02 to 0.03 |
|  |  | AA | -0.0008 |  | -0.02 to 0.02 |
| rs363504-rs363538 | IA (DSM) | C-A | -0.003 | 1.87 (0.59) | -0.01 to 0.009 |
|  |  | C-C | 0.05 |  | -0.04 to 0.14 |
|  |  | T-A | 0.004 |  | -0.006 to 0.01 |
|  |  | T-C | 0.004 |  | -0.008 to 0.01 |
|  | BPr (CPRS) | C-A | -0.001 | 0.76 (0.85) | -0.01 to 0.01 |
|  |  | C-C | 0.02 |  | -0.03 to 0.07 |
|  |  | T-A | 0.002 |  | -0.01 to 0.01 |
|  |  | T-C | 0.004 |  | -0.01 to 0.01 |
|  | IA (CPRS) | C-A | -0.003 | 1.84 (0.59) | -0.01 to 0.009 |
|  |  | C-C | 0.05 |  | -0.04 to 0.14 |
|  |  | T-A | 0.005 |  | -0.006 to 0.01 |
|  |  | T-C | 0.004 |  | -0.008 to 0.01 |
|  | HA (CPRS) | C-A | -0.007 | 1.90 (0.59) | -0.02 to 0.004 |
|  |  | C-C | 0.003 |  | -0.02 to 0.03 |
|  |  | T-A | 0.007 |  | -0.004 to 0.01 |
|  |  | T-C | 0.005 |  | -0.006 to 0.02 |
|  | AI (CPRS) | C-A | -0.004 | 0.92 (0.82) | -0.02 to 0.007 |
|  |  | C-C | 0.01 |  | -0.03 to 0.06 |
|  |  | T-A | 0.005 |  | -0.007 to 0.02 |
|  |  | T-C | 0.006 |  | -0.007 to 0.02 |
